# Differential miRNA expression profiles in Peruvian HTLV-1 carriers

**DOI:** 10.1186/1742-4690-11-S1-P128

**Published:** 2014-01-07

**Authors:** Jason Rosado, Carolina Alvarez, Daniel Clark, Eduardo Gotuzzo, Michael Talledo

**Affiliations:** 1 Laboratorio de Epidemiología Molecular, Universidad PeruanaCayetano Heredia, Lima, Peru; 2 Instituto de Medicina Tropical Alexander von Humboldt, Universidad PeruanaCayetano Heredia, Lima, Peru; 3 Rega Institute for Medical Research, Katholieke Universiteit Leuven, Leuven, Belgium; 4 Laboratorios de Investigación y Desarrollo, Universidad Peruana Cayetano Heredia, Lima, Peru; 5 Facultad de Medicina, Universidad PeruanaCayetano Heredia, Lima, Peru; 6 Hospital Nacional Cayetano Heredia, Lima, Peru

## 

MicroRNAs (miRNAs) are small non-coding RNAs that regulate protein expression. HTLV-1 is able to promote oncogenesis in T cells by altering the expression of miRNAs involved in the control of cell-cycle. It is not known whether HTLV-1 deregulates miRNAs expression in cells of HTLV-1-associated myelopathy/tropical spastic paraparesis (HAM/TSP) patients. To asses if HTLV-1 infection might alter the expression of miRNAs involved in inflammatory response, we evaluated the expression of 84 miRNAs involved in inflammatory process in asymptomatic HTLV-1 carriers (AC) and HAM/TSP patients using the miScript miRNA PCR Array Human Inflammatory Response & Autoimmunity (SABioscience). For this purpose, fourteen HTLV-1-positive individuals were selected and classified into three groups: five asymptomatic carriers (AC), 4 HAM/TSP patients with EDSS score of 1-5 (=mild HAM/TSP), and 5 HAM/TSP patients with EDSS score of 5.5-9 (=severe HAM/TSP). Total RNA was isolated from PBMCs and pooled according to the groups. qBase software was used for normalization, ANOVA was used for comparisons and False Dicosvery Rate to correct for multiple comparisons. We found nine differentially expressed miRNAs between AC and HAM/TSP patients (mild and severe HAM/TSP). Twelve miRNAs were differentially expressed among mild HAM/TSP, severe HAM/TSP and AC groups. These findings support results previously reported in Adult T-cell leukemia/lymphoma (ATLL) cells, in which hsa-miR-145, miR-130a, miR181a and miR101a were found to be down-regulated and miR-30d was found to be up-regulated in comparison to those of healthy donors. Further analysis to confirm these findings are needed.

